# Marine Chitosan-Oligosaccharide Ameliorated Plasma Cholesterol in Hypercholesterolemic Hamsters by Modifying the Gut Microflora, Bile Acids, and Short-Chain Fatty Acids

**DOI:** 10.3390/nu15132923

**Published:** 2023-06-27

**Authors:** Abdullah Abdo, Chengnan Zhang, Sam Al-Dalali, Yakun Hou, Jie Gao, Mohammed Abdo Yahya, Ali Saleh, Hamzah Aleryani, Zakarya Al-Zamani, Yaxin Sang

**Affiliations:** 1 College of Food Science and Technology, Hebei Agricultural University, Baoding 071001, China; drabdoabdullah2021@gmail.com (A.A.); yakunhou86@hotmail.com (Y.H.); gaojiehbu@163.com (J.G.);; 2 Department of Food Sciences and Technology, Faculty of Agriculture and Food Sciences, Ibb University, Ibb 70270, Yemen; salihsam4@gmail.com (S.A.-D.); aliqashaab@gmail.com (A.S.); keminmao@outlook.com (Z.A.-Z.); 3 School of Food Science and Health, Beijing Technology and Business University, Beijing 100048, China; zhangcn@btbu.edu.cn; 4 Department of Food Science and Nutrition, College of Food and Agriculture Sciences, King Saud University, Riyadh 11451, Saudi Arabia; mabdo@ksu.edu.sa

**Keywords:** chitosan-oligosaccharide, plasma cholesterol, gut microflora, bile acids, short-chain fatty acids

## Abstract

This study evaluated the cholesterol-alleviating effect and underlying mechanisms of chitosan-oligosaccharide (COS) in hypercholesterolemic hamsters. Male hamsters (*n* = 24) were divided into three groups in a random fashion, and each group was fed one particular diet, namely a non-cholesterol diet (NCD), a high-cholesterol diet (HCD), and an HCD diet substituting 5% of the COS diet for six weeks. Subsequently, alterations in fecal bile acids (BAs), short-chain fatty acids (SCFAs), and gut microflora (GM) were investigated. COS intervention significantly reduced and increased the plasma total cholesterol (TC) and high-density lipoprotein-cholesterol (HDL-C) levels in hypercholesteremic hamsters. Furthermore, Non-HDL-C and total triacylglycerols (TG) levels were also reduced by COS supplementation. Additionally, COS could reduce and increase food intake and fecal SCFAs (acetate), respectively. Moreover, COS had beneficial effects on levels of BAs and GM related to cholesterol metabolism. This study provides novel evidence for the cholesterol-lowering activity of COS.

## 1. Introduction

A high level of blood cholesterol is usually considered the key risk factor for heart disease [1]. Therefore, the successful control of blood cholesterol is considered an effective approach to ameliorating heart disease occurrence [2]. Currently, there is an increasing demand to develop new drugs that can maintain low plasma TC levels [3]. Though the TC-lowering drugs available in the market are quite effective, their synthetic chemical nature can pose serious negative effects on consumers, which can result in adverse consequences [4,5]. Given the side effects of the existing TC medicines, natural bioactive compounds are considered an alternative approach to TC-lowering agents, which remains an urgent necessity.

As a natural bioactive compound, it was stated that marine chitosan-oligosaccharide (COS) has the ability to alleviate liver diseases by improving dyslipidemia in obese rats [3,6,7]. A recent report showed that dietary chitin oligosaccharides, a chitosan derivative, could markedly lower blood TC levels in atherosclerosis-prone apolipoprotein E-deficient (Apoe^−/−^) mice [8]. In diet-induced obese rats, supplementation of COS into a diet could improve dyslipidemia via reducing TC and TG levels [6]. However, the results from other animal and human experiments were shown to be inconsistent. It was found that a diet with a COS supplement could not affect LDL-C in rats fed a high-fat diet [9]. In patients with hypercholesterolemia, the HDL-C, LDL-C, and TG levels remained unchanged by COS consumption [10]. Therefore, further studies are needed to establish the anti-hypercholesterolemia activity of COS. Although the mechanism of COS’s effect on blood cholesterol is not yet fully understood, a few studies have proposed that COS’s effect on blood cholesterol is likely mediated by regulating BA-related enzymes and genes [7].

In cholesterol homeostasis, GM participates in fermenting indigestible food components and producing SCFAs, which are effective in lowering blood cholesterol [11,12,13]. In addition, GM plays a crucial role in BAs’ metabolism. Approximately 90% of conjugated and a small percentage of unconjugated BAs will return to the liver via enterohepatic circulation after being reabsorbed in the distal ileum. The GM deconjugates a small fraction of conjugated BAs as they travel toward the ileum [14]. Diminishing the conjugated BAs and increasing the unconjugated BAs will decrease reabsorbed BAs and blood TC [13]. Therefore, fecal BAs should be summed up to reflect BA metabolism. In this regard, COS had beneficial action on altering the GM and SCFAs production in mice [15,16]. Nevertheless, how COS prevents a high cholesterol diet-induced hypercholesterolemia, especially the improvements in BAs and GM, remains largely unclear.

In cholesterol studies, hamsters are usually utilized as human surrogates as they closely mimic the metabolic characteristics of human cholesterol. To the best of our knowledge, the effect of COS in hypercholesterolemic hamsters is still unclear. Thus, this research was conducted to evaluate the cholesterol-alleviating action of marine chitosan-oligosaccharide in hypercholesterolemic hamsters and investigate the bile acids, SCFAs, and intestinal microbiota alteration in relation to COS’s cholesterol-alleviating action. In this study, the effect of COS on GM and the BA profiles of hamsters fed high cholesterol time were evaluated for the first time.

## 2. Materials and Methods

### 2.1. Materials

Chitosan-oligosaccharide was given by Qingdao BZ Oligo Biotech Co., Ltd. (Qingdao, China). COS (91.2% degree of deacetylation, average molecular weight of 1231 Da) with 92.3% purity was produced by enzymatic hydrolysis from the chitosan of crab shells. The standards, chemicals, and kits were purchased from Sigma-Aldrich (St. Louis, MO, USA).

### 2.2. Diets

NCD, HCD, and COS diets were produced by Trophic Animal Feed High-tech Co., Ltd. (Nantong, China). An NCD was produced by mixing the components, as shown in Table 1. An HCD was prepared by mixing the NCD diet with cholesterol (0.2%). The 0.2% cholesterol used in this study to induce hypercholesterolemia in hamsters was based on a previous report [17] A COS was prepared by mixing the HCD diet with 5% COS (Table 1). The quantity of COS used in this work was equivalent to 19 g/day daily consumption for human based on humans consume 2000 kcal/day and physically achievable for adults [18].

### 2.3. Hamsters

Twenty-four male hamsters (8 weeks old) were purchased from Beijing Vital River Laboratory Animal Technology Co., Ltd. (Beijing, China) and housed in a room (*n* = 2 per cage) at 23 °C with changing light for twelve hours. The hamsters were acclimated for 2 weeks before being randomly divided into three groups (*n* = 8 each) and fed for 6 weeks on one of the three diets (NCD, HCD, and COS). The hamster’s blood was collected at weeks 0 and 6. Food intake and body weight were recorded every two days and one week, respectively. After 6 weeks, the hamsters were sacrificed, and the organs were collected, washed, and stored at −80 °C until analysis. Hamsters have been cared for according to the guidelines of the national health institutes (NIH Publications No. 8023, revised 1978) and institutional protocols (serial No. UJS-LAER-2018042301). The experimental guidelines were approved by the Animal Experimental Ethical Committee, Beijing Sport University (Reference number: 2019008A).

### 2.4. Plasma Lipid Measurements

TC, high-density lipoprotein-cholesterol (HDL-C), and total triacylglycerols (TG) in hamsters’ plasma were detected using a biochemical analyzer (AU5800, Beckman Coulter Co., Ltd., Brea, CA, USA) by following the procedure of commercial kits (Zhongshengbeikong Co., Ltd., Beijing, China). Non-HDL-C was determined by deducting HDL-C from TC.

### 2.5. BAs Measurements

Briefly, BAs in 100 mg of fecal samples were extracted using 1 mL of methanol. BAs in the optioned supernatant after 30 min of centrifugation at 12,000 rpm were analyzed. BAs were separated using a Dionex™ UltiMate™ 3000 Rapid Separation LC (RSLC) system (Thermo Scientific, Waltham, MA, USA) equipped with an HSS T3C18 column (2.1 mm × 100 mm, 1.8 μm, Waters, Milford, CT, USA). The injection volume, temperature, and flow rate were set to 50 °C, 10 μL, and 300 μL/min, respectively. Methanol (A) and 2 mmol/L of ammonium acetate (B) were used as the mobile phases. Furthermore, the mass spectrometry was performed using a Thermo Scientific TM Q Exactive TM hybrid quadrupole Orbitrap mass spectrometer equipped with a HESI-II probe. The negative HESI-II spray voltage was 3.5 kV. The heated capillary temperature, sheath gas pressure, auxiliary gas setting, and heated vaporizer temperature were 320 °C, 30 psi, 10 psi, and 300 °C, respectively. The auxiliary gas, sheath gas, and collision gas were all nitrogen at 1.5 m Torr. The full mass scan parameters were set as follows: an auto gain control target under 1 × 10^6^, a resolution of 70,000, an *m*/*z* range of 150–1500, and a maximum isolation time of 50 ms [19].

### 2.6. Measurement of Fecal’s SCFAs and pH Values

The SCFAs in freeze-dried fecal samples were extracted by 0.1 mol/L of PBS buffer (pH 7.4) and analyzed using Agilent 1260 UV High-Performance Liquid Chromatography equipped with a UV detector and Aminex HPX-87H column (Bio-Rad Laboratories, Hercules, CA, USA), respectively. SCFAs were separated using 0.005 N of sulfuric acid. pH values in fecal samples were measured using a pH meter (Mettler Toledo, Zurich, Switzerland) after dilution with distilled water [19,20].

### 2.7. Measurement of Intestinal Microflora

Total bacteria’s DNA in fresh feces was extracted and measured using the QIAamp^®^ DNA Mini Kit (Qiagen, Valencia, CA, USA) and the Nano-Drop 1000 spectrophotometer (Nano-Drop Technologies, Wilmington, DE, USA), respectively. The regions of the 16S rRNA gene in V3-V4 were amplified using commercial forward primer 338 F (5′-ACTCCTACGGGAGGCAGCA-3′) and the reverse primer 806 R (5′-GGACTACHVG GGTWTCTAAT-3′). After purification of amplicons using the AxyPrep DNA gel extraction kit (Axygen Biosciences, Union City, CA, USA) and quantification using QuantiFluor™-ST (Promega, Madison, WI, USA), the sequencing was performed at Shanghai Majorbio Bio-pharm Technology Co., Ltd. (Shanghai, China) on a MiSeq platform (Illumina, San Diego, CA, USA) [19].

### 2.8. Data Analysis

SPSS software (version 25.0, SPSS Inc., Chicago, IL, USA) was used to perform a one-way analysis of variance (ANOVA) on the data. The significant changes were determined as the *p*-value was less than 0.05. Spearman’s correlation was performed to find the correlations between parameters of cholesterol metabolism and relative genus abundances. A free online platform of Majorbio’s I-Sanger (www.i-sanger.com) was used to analyze the microbiome data after normalization on 15 September 2022. Alpha diversity was assessed by computing Simpson and Shannon indexes. The rationality and efficiency of the sequencing depth were determined using rarefaction analysis. Beta diversity was estimated by partial least squares discriminant analysis (PLS-DA) and by hierarchical cluster analysis. The data were analyzed on the free online platform of Majorbio I-Sanger Cloud Platform (www.i-sanger.com) on 15 September 2022. Values were expressed as an average ± standard deviation (SD).

## 3. Results

### 3.1. Food Intake, Energy Intake, and Body and Organ Weights

The energy and food intake of the COS hamsters were significantly (*p* < 0.05) slower than those of the HCD hamsters, suggesting that COS may decrease the hamster’s appetite. At week 6, COS reduced the body weight of hypercholesteremic hamsters, but COS did not significantly change the body weight. COS also had no significant effect on organ weights, except that the weight of epididymal fat in COS hamsters was significantly (*p* < 0.05) smaller than that of the HCD and NCD hamsters (Table 2).

### 3.2. Plasma Lipid Profile

As revealed in Table 3, the hamsters fed a 0.2% cholesterol diet for 6 weeks significantly elevated plasma TC and non-HDL-C compared with those fed an NCD diet, indicating that the hamster’s experimental hypercholesterolemia model was successful. The elevated plasma TC in HCD-diet-fed hamsters was successfully (*p* < 0.05) reversed by 5% COS supplementation. Non-HDL-C and TG levels were also decreased by COS. Similarly, plasma HDL-C in the NCD and COS groups was higher (*p* < 0.05) than that of the HCD group.

### 3.3. Fecal BAs

Briefly, The COS diet could reverse the high-cholesterol-induced BA alteration. Compared with HCD, COS supplementation led to an increase in the excretion of unconjugated fecal BAs, including allocholic acid (AlloCA), cholic acid (CA), gamma-muricholic acid (*γ*-MCA), and deoxycholic acid (DCA). Excretion of other unconjugated BAs was also increased by COS feeding, but there was no significant alteration in comparison with the HCD group. In contrast, COS supplementation significantly decreased conjugated BAs, including glycolithocholic acid (GLCA), glycohyodeoxycholic acid (GHDCA), taurolithocholic acid (TLCA), taurochenodeoxycholic acid (TCDCA), and taurodeoxycholic acid (TDCA), which were significantly (*p* < 0.05) decreased by COS supplementation, indicating that conjugated BAs were converted into unconjugated BAs (Table 4).

### 3.4. Fecal SCFA Contents and pH Value

The fecal SCFA analysis found the major SCFAs were butyrate and acetate (10.283 ± 1.776 mg/day/hamster), followed by the minor propionate in fecal NCD hamsters (Table 5). On the one hand, COS could significantly (*p* < 0.05) enhance acetate production in HCD hamsters. On the other hand, the generation of propionate and butyrate, as well as total SCFAs, were also increased, but they were not significantly (*p* < 0.05) change by the addition of COS (Table 5). Results displayed that pH values of feces in the hamster groups were in the order of HCD > COS > NCD (Table 5). In short, hamsters fed with COS had a markedly (*p* < 0.05) lower fecal pH value than the HCD group.

### 3.5. Overall Structure and Composition of GM

To evaluate the influence of dietary COS on GM composition, the GM in fresh fecal hamsters was determined. The GM by the administration of COS was revealed by analyzing the structure and composition of gut bacteria.

**Figure 1:**
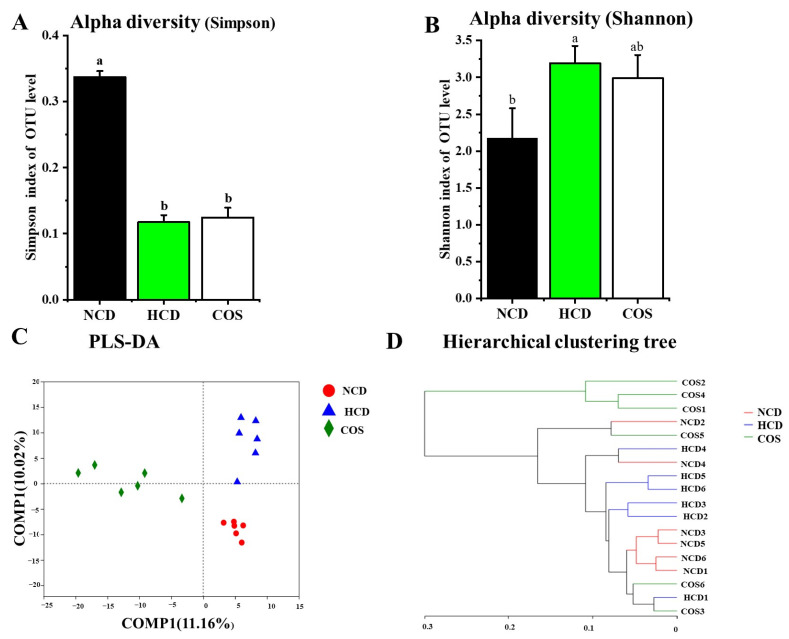
Effect of COS supplementation on gut flora diversity in hamsters. Alpha diversity assessed by using a Simpson index (**A**) and Shannon index (**B**) Beta diversity assessed by using PLS-DA analysis (**C**) and hierarchical clustering tree (**D**) on OTU level. NCD, non-cholesterol diet; HCD, high-cholesterol diet; COS, HCD with addition of 5% chitosan-oligosaccharide (COS). Values were expressed as mean ± SD. ^a^ and ^b^ Means at the same row with different letters differed significantly at *p* < 0.05 by one-way ANOVA analysis.

GM diversity was decreased by COS addition, including the increased Simpson index and the decreased Shannon index at the OUT level (Figure 1A,B). Furthermore, the PLS-DA analysis showed a distinct divergence in GM composition between the three groups (Figure 1C). Hierarchical clustering tree results showed that COS was separated from HCD (Figure 1D).

According to the Simpson and Shannon indexes, the rarefaction curve has flattened over time, indicating that most of the flora diversity has been retrieved from a sufficient volume (Figure S1).

**Figure 2:**
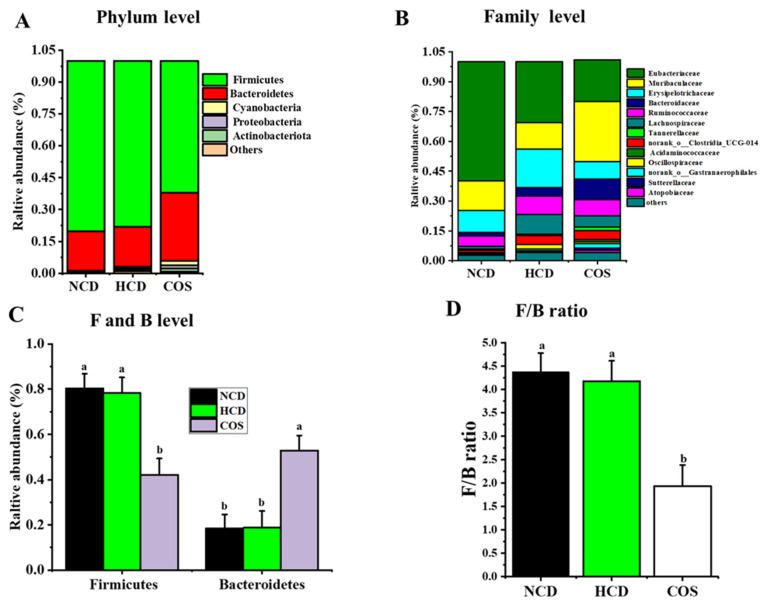
Effect of COS supplementation on gut flora composition in hamsters at phylum and family levels. Composition of gut flora at phylum level (**A**), family level (**B**), the relative abundance of Firmicutes and Bacteroidetes (**C**), and the ratio of Firmicutes/Bacteroidetes (**D**) NCD, non-cholesterol diet; HCD, high-cholesterol diet; COS, HCD with addition of 5% chitosan-oligosaccharide (COS). Values were expressed as mean ± SD. ^a^ and ^b^ Means at the same row with different letters differed significantly at *p* < 0.05 by one-way ANOVA analysis.

**Figure 3:**
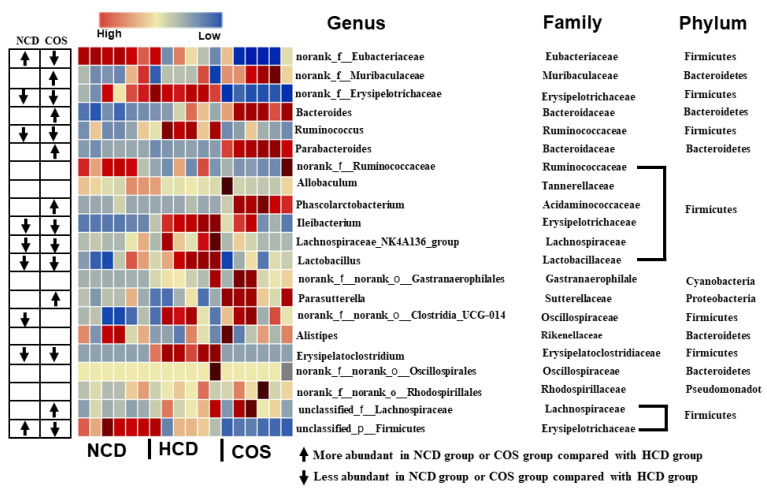
Effect of COS supplementation on gut flora composition in hamsters at genus level. NCD, non-cholesterol diet; HCD, high-cholesterol diet; COS, HCD with addition of 5% chitosan-oligosaccharide (COS).

The compositions of gut bacteria are shown in Figure 2 and Figure 3. At the phylum level, hamsters fed with COS had a significant increase in the number of *Bacteroidetes*, whereas they had a significant decrease in the number of *Firmicutes* and *Firmicutes/Bacteroidetes* ratio (F/B) in comparison with HCD hamsters (Figure 2A,D).

At the family level, Eubacteriaceae, Muribaculaceae, Erysipelotrichaceae, Ruminococcaceae, Bacteroidaceae, Lachnospiraceae, and Tannerellaceae represented the most abundant families (Figure 2B). The numbers of Muribaculaceae and Bacteroidaceae in COS were higher than that of the HCD group, while the concentrations of Erysipelotrichacea, Eubacteriaceae, and Ruminococcaceae were alleviated in the COS group (Figure 2B).

At the genus level, it was found that *norank_f_Eubacteriaceae*, *norank_f_Muribaculaceae*, *norank_f_Erysipelotrichaceae, Ruminococcus, Bacteroides*, and *Parabacteroides* were the most abundant genera. In particular, the levels of SCFA-producing bacteria in COS, including *norank_f_Muribaculaceae, Bacteroides, Parabacteroides*, and *Parasutterella,* were significantly higher than those in the HCD group, while COS significantly reduced the phylum of *Firmicutes*, including *norank_f_Eubacteriaceae*, *norank_f_Erysipelotrichaceae*, and *Ruminococcus* (Figure 3).

### 3.6. Spearman Correlation

**Figure 4:**
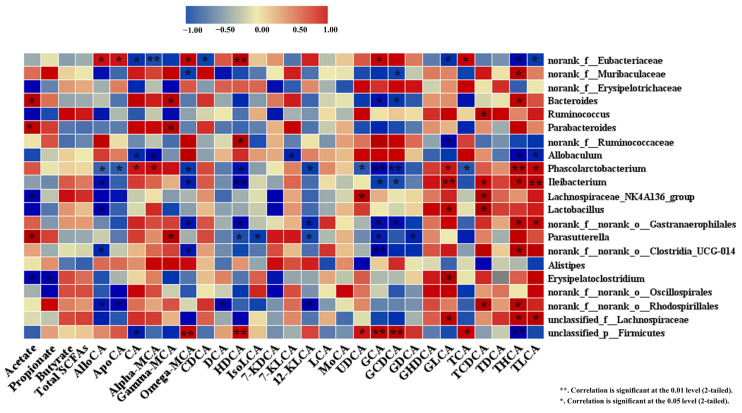
Spearman’s correlation analysis between 21 key genera and SCFAs and bile acids in hamsters fed one of three diets: NCD, non-cholesterol diet; HCD, high-cholesterol diet; COS, HCD with addition of 5% chitosan-oligosaccharide (COS). The red to blue in the heatmap represented the R value of spearman’s correlation changed from greater to lower. AlloCA, allocholic Acid; ApoCA, apocholic acid; CA, cholic acid; Alpha-MA, α muricholic acid; Gamma-MA; γ-muricholic Acid; Omega—MA, ω-muricholic acid; CDCA, chenodeoxycholic acid; DCA, deoxycholic acid; HDCA, hyodeoxycholic acid; IsoLCA, isoalblithocholic acid; 7-KDCA,7-ketodeoxycholic acid; 7-KLCA, 7-ketolithocholic acid; 12-KLCA, 12-ketolithocholic acid; LCA, lithocholic acid; MoCA, murocholic acid; UDCA, ursodeoxycholic acid; GCA, glycocholic acid; GCDCA, glycochenodeoxycholic acid; GDCA, glycodeoxycholic acid; GHDCA, glycohyodeoxycholic acid; GLCA, glycolithocholic acid; TCA, taurocholic acid; TCDCA, taurochenodeoxycholic acid; TDCA, taurodeoxycholic acid; THCA, taurohyodeoxycholic acid; TLCA, taurolithocholic acid.

Spearman’s correlation analysis examined the association between the modification of GM and cholesterol-related markers. In comparison between the most abundant genera, *norank_f_Erysipelotrichaceae* showed a negative correlation with acetate and unconjugated *γ*-MCA, while *Bacteroides* and *Parabacteroides* displayed a positive association with acetate, and unconjugated *γ*-MCA and only *Bacteroides* presented a negative correlation with conjugated glycocholic acid and glycochenodeoxycholic acid (GCDCA) and taurocholic acid (TCA). In this study, we found that *norank_f_Muribaculaceae* was correlated positively with unconjugated *γ*-MCA and negatively with conjugated GCDCA, while *norank_f_Eubacteriaceae* was correlated negatively with CA, Alpha-CA, GLCA, Taurohyodeoxycholic acid (THCA), and TLCA and positively with AlloCA, apocholic acid, HDCA, and TCA, as presented in Figure 4.

## 4. Discussion

Although dietary cholesterol has a controversial effect on plasma TC, researchers found a close correlation between high TC levels and heart disease [21,22,23]. In this study, the dietary addition of COS at 5% was significantly effective in ameliorating TC in plasma hypercholesteremic hamsters (Table 3). The amount of COS used in present study was equivalent to 19 g/day daily based on human consumption of 2000 kcal/day which is physically achievable for an adult [18]. Our result was in agreement with the result of a previous study [7]. In their study, plasma cholesterol was lowered in rats fed a high-fat diet with an added 5% COS [7]. In hyperlipidemic rats, COS remarkably lowered cholesterol by promoting BA-related enzymes [3]. To our knowledge, alleviation of TC in hamsters fed high cholesterol diet by COS was reported for the first time in our study. However, COS in our study had a significant effect on improvement of plasma lipids, which was consistent with previously reports [6,7].

In line, we found that the food intake in the COS group was significantly less than that of the HCD group, suggesting the TC-lowering effect of COS is in part mediated by reducing cholesterol intake (Table 2). Similarly, COS could reduce food intake in obese mice [24]. Such a result may be related to the ability of COS to modulate appetite-related hormones. It was reported that chitosan (source of COS) could successfully modulate appetite-related hormones such as adiponectin and leptin [25].

The major pathway for reducing blood cholesterol is by enhancing its conversion to BAs [19,26,27]. Therefore, we compared the excretion of fecal BAs among the three groups. We found that the HCD diet had altered levels of several individuals of BAs in feces compared with the NCD diet, whereas the COS diet could moderately reverse the high cholesterol-induced alteration (Table 5), indicating that the cholesterol-alleviating effect of COS was partially regulated via improving BA synthesis. In particular, COS could reduce levels of several conjugated BAs, including T-DCA, THCA, TLCA, TCDCA, GLCA, and GHDCA, while increasing the number of unconjugated BAs, such as CA, *γ*-MCA, AlloCA, and DCA (Table 4). These results were in agreement with earlier findings stating that COS could regulate BA synthesis-related enzymes [3]. It was reported that COS could reduce plasma cholesterol in rats via upregulating the activity of cholesterol 7α-hydroxylase (CYP7A1), which assists in conversion of cholesterol into bile acids [3]. This enzyme is produced by gut flora, especially Bacteroides and Eubacterium [28]. What is more, a small fraction of conjugated bile acids is deconjugated by gut microbiota as they travel down toward the ileum [14]. In this regards, reducing the ratio of conjugated BAs to unconjugated BAs will result in a decrease in the reabsorption of BAs as well as plasma TC [13]. Thus, the enhanced excretion of unconjugated BAs in feces could be related to hydrolysis of conjugated BAs via bile salt hydrolases (BSHs). This enzyme is produced by gut bacteria such as *Bacteroides* spp., *Listeria*, and *Brucella*. *Bacteroides* spp. especially plays a major role in deconjugation of conjugated BAs [29]. TC conversion into BAs can be promoted by SCFAs [13,30]. In our study, COS could enhance SCFA-generating bacteria and BA synthesis bacteria. Thus, the gut microbiota is essential for synthesis and *excretion* of *BAs.* In connection with this finding, reducing the intestine pH may also boost the hydrolysis of conjugated BAs to unconjugated BAs and improve the excretion of unconjugated BAs [13]. COS could obviously reduce the fecal pH value of HCD hamsters, leading to enhanced conjugated BA hydrolysis into unconjugated BAs. Secondary BAs are less well absorbed and mostly secreted in the feces, leading to loss of plasma TC [31]. Thus, BA synthesis and deconjugation are an effective way to regulate of cholesterol in the body, by taking cholesterol out of the circulation to be used for the synthesis of new BAs, replacing those lost in the feces [31]. To our knowledge, it is the first report that COS is able to influence the BA profile in hamsters fed with a high-cholesterol diet.

Another mechanism connected to the TC-ameliorating ability of COS was partially attributable to its effect on SCFA generation by GM. SCFAs are capable of diminishing blood TC by promoting the conversion of cholesterol into BAs [13,30]. Dietary supplementation with COS markedly enhanced acetate production (Table 5). The ability of COS to promote SCFA production has previously been reported, which is consistent with our findings [8]. This suggests that COS’s TC-ameliorating ability was accompanied by promoting SCFA production by increasing the levels of SCFA-producing bacteria. Thus, the population of microflora in the gut was investigated to prove this hypothesis. The present work showed that COS diets could alter the composition of the GM in HCD hamsters by lowering and boosting *Firmicutes and Bacteroidetes*, respectively, leading to a notable reduction of the F/B ratio (Figure 2D). The F/B ratio is positively linked with cardiovascular diseases and obesity [27]. Similarly, COS could increase *Bacteroidetes* abundance at the expense of *Firmicutes* and lower the F/B ratio in colitis mice [32].

*Muribaculaceae* and *Bacteroidaceae* enrichments in the COS groups were observed at the family level (Figure 2B), which are SCFA-producing bacteria [33,34,35]. Accompanied by this observation, *Erysipelotrichaceae* in HCD hamsters was reduced by COS. Numerous studies have shown that the *Erysipelotrichaceae* level was positively and negatively correlated with increased liver cholesterol and fecal cholesterol excretion, respectively [36]. At the genus level, COS could elevate the relative concentration of SCFA-generating bacteria, including *norank_f_Muribaculaceae*, *Bacteroides*, and *Parabacteroides* [15,37]. Previously, *norank_f_Muribaculaceae*, *Bacteroides*, and *Parabacteroides* were increased by COS treatment [32,38,39], which was consistent with our study. In connection with this, SCFA-generating bacteria, especially *Bacteroides*, play a crucial role in BA synthesis and excretion as mentioned above. COS could reduce *norank_f_Erysipelotrichaceae*, a bacteria linked to fat (cholesterol) accumulation in the human liver [40]. On this point, a reduction in *norank_f_Erysipelotrichaceae* connected with feeding COS diets may contribute to the cholesterol-ameliorating ability of COS detected in the plasma.

In addition, results from Spearman’s correlation analysis showed that improvement in acetate generation was positively associated with a rise in relative abundance of *Bacteroides* and *Parabacteroides* and negatively associated with *norank_f_Erysipelotrichaceae* (Figure 4). These results indicate the hypocholesterolemic activity of COS was in part mediated by shifting GM composition and promoting SCFAs production. GM also plays a vital role in regulating the type and quantity of BAs via promoting and modulating BA synthesis *excretion* [2]. In the correlation analysis, the alterations in several BAs were associated with modifications in the key GM genera (Figure 4). However, the effect of COS on the gut flora of hypercholesterolemia hamsters was studied for the first time in this work. In short, COS induced beneficial BA modification, which was associated positively with the abundance of *norank_f_Muribaculaceae*, *Bacteroides*, and *Parabacteroides* in the gut. Our results suggested that the alteration in the GM was attributable to the cholesterol-lowering effect of COS via improved SCFA and BA generation.

## 5. Conclusions

This study revealed that dietary supplementation with 5% COS-alleviated hyperchloremia in hamsters via reducing food intake, SCFA generation, and alteration of GM and BAs. The favorable GM-regulatory activity was characterized by reducing *Firmicutes* and increasing *Bacteroidetes* and SCFA-producing bacteria. Dietary supplementation with COS could reduce plasma TC via enhancement of fecal excretion of bile acids. Such benefit was accompanied by improving SCFA generation and remodeling gut flora. This work may provide valuable evidence for future clinical trials of COS for hypercholesterolemic subjects.

## Figures and Tables

**Table 1:** Compositions of three diets: NCD, non-cholesterol diet; HCD, high-cholesterol diet; COS, HCD with addition of 5% chitosan-oligosaccharide (COS).

	NCD	HCD	COS
Ingredients			
Corn starch	50.9	50.9	50.9
Sucrose	11.9	11.9	11.9
Lard	5	5	5
AIN 93 M Mineral Mix	4	4	4
AIN 93 Vitamin Mix	2	2	2
Gelatin	2	2	2
Cholesterol	0	0.2	0.2
Casein	24.2	24.2	24.2
Chitosan-oligosaccharide	0	0	5
Total (g)	100	100.2	105.2
Energy %			
Protein	26	26	26
Carbohydrates	63	63	63
Fat	11	11	11
Total (kcal)	401	401	401

**Table 2:** Food intake, energy intake, and body and organ weights in hamsters fed one of the three diets: NCD, non-cholesterol diet; HCD, high-cholesterol diet; COS, HCD with addition of 5% chitosan-oligosaccharide (COS).

	NCD	HCD	COS	*p* Value
Food intake (g/hamster/day)	10.481 ± 0.174 ^c^	10.924 ± 0.145 ^a^	10.451 ± 0.254 ^bc^	0.031
Energy intake	40.877 ± 0.681 ^b^	43.698 ± 0.581 ^a^	39.714 ± 0.965 ^b^	0.001
(kcal/hamster/day)				
Body weights (g)				
week 0	142.712 ± 10.302	137.887 ± 12.922	141.375 ± 7.448	0.771
week 6	147.725 ± 18.378	149.5 ± 13.318	139.425 ± 14.41	0.233
Organ weights (g)				
Liver	5.5453 ± 1.030 ^b^	7.707 ± 1.189 ^a^	7.336 ± 1.973 ^a^	0.005
Heart	0.65 ± 0.068	0.66 ± 0.064	0.635 ± 0.076	0.932
Kidney	1.216 ± 0.244	1.27 ± 0.097	1.37 ± 0.09	0.343
Testis	4.046 ± 0.595	4.511 ± 0.686	4.136 ± 0.434	0.472
Epididymal fat	2.882 ± 0.713 ^ab^	3.807 ± 0.806 ^a^	2.158 ± 1.192 ^b^	0.006
Perirenal fat	1.205 ± 0.485	1.43 ± 0.386	1.001 ± 0.513	0.12

Values were expressed as mean ± SD (*n* = 8). ^a,b,c^ Means at the same row with different letters differed significantly at *p* < 0.05.

**Table 3:** Plasma total cholesterol (TC), triacylglycerol (TG), high-density lipoprotein cholesterol (HDL-C), and non-HDL-C in hamsters fed one of three diets: NCD, non-cholesterol diet; HCD, high-cholesterol diet; COS, HCD with addition of 5% chitosan-oligosaccharide (COS).

	NCD	HCD	COS	*p* Value
Week 0				
TC (mmol/L)	4.047 ± 1.816	4.142 ± 0.749	4.103 ± 0.443	0.992
HDL-C (mmol/L)	3.081 ± 0.448	3.057 ± 0.549	3.007 ± 0.365	0.192
Non-HDL-C (mmol/L)	0.966 ± 0.120	1.582 ± 0.328	1.598 ± 0.175	0.597
TG (mmol/L)	1.476 ± 0.734	1.598 ± 0.593	1.512 ± 0.709	0.192
Week 6				
TC (mmol/L)	5.076 ± 0.396 ^c^	7.897 ± 0.910 ^a^	6.901 ± 0.601 ^b^	<0.001
HDL-C (mmol/L)	3.430 ± 0.258 ^a^	2.503 ± 0.733 ^b^	3.245 ± 0.735 ^a^	<0.001
Non-HDL-C (mmol/L)	1.646 ± 0.405 ^c^	5.393 ± 0.607 ^a^	3.656 ± 1.063 ^b^	<0.001
TG (mmol/L)	1.578 ±0.257 ^b^	2.602 ± 0.421 ^a^	1.793 ± 0.594 ^b^	<0.001

Values were expressed as mean ± SD (*n* = 8). ^a,b,c^ Means at the same row with different letters differed significantly at *p* < 0.05.

**Table 4:** Fecal bile acids quantity at week 6 in hamsters fed one of three diets: NCD, non-cholesterol diet; HCD, high-cholesterol diet; COS, HCD with addition of 5% chitosan-oligosaccharide (COS).

Bile Acids (µg/Day/Hamster)	NCD	HCD	COS	*p* Value
Unconjugated				
Allocholic Acid	85.915 ± 7.709 ^a^	0.605 ± 0.092 ^b^	72.168 ± 2.520 ^a^	<0.001
Apocholic acid	140.871 ± 23.196	89.621 ± 1.84	110.233 ± 8.352	0.126
Cholic acid	20.578 ± 1.550 ^a^	5.892 ± 2.461 ^b^	18.372 ± 3.245 ^a^	<0.001
Alpha-muricholic acid	1.838 ±0.085 ^b^	2.765 ± 0.567 ^a^	8.238 ± 0.698 ^a^	<0.001
Gamma -muricholic Acid	3.094 ± 0.748 ^b^	7.269 ± 2.367 ^b^	21.244 ± 4.853 ^a^	<0.001
Omega -muricholic acid	496.639 ±24.622 ^a^	282.820 ± 8.018 ^b^	384.075 ± 16.634 ^ab^	<0.001
Chenodeoxycholic acid	97.371 ± 4.579	121.613 ± 8.430	157.724 ± 21.487	0.465
Deoxycholic acid	1200.654 ± 89.140 ^a^	651.065 ± 80.756 ^b^	916.169 ± 75.202 ^a^	<0.001
Hyodeoxycholic acid	27.534 ± 1.795 ^a^	9.880 ± 0.606 ^b^	19.203 ± 1.705 ^a^	<0.001
Isoalblithocholic acid	683.351 ± 31.117	453.915 ± 25.199	683.726 ± 20.337	0.136
7-ketodeoxycholic acid	33.260 ± 5.170	32.864 ±3.13	47.268 ± 9.721	0.159
7-ketolithocholic acid	7.972 ± 1.827	11.809 ± 2.694	14.670 ± 4.548	0.179
12-ketolithocholic acid	428. 92 ± 40.582	255.830 ± 60.334	318.718 ± 17.268	0.064
Lithocholic acid	1158.628 ± 107.725	1081.634 ± 101.04	1333.827 ± 103.588	0.133
Murocholic acid	13.782 ± 4.008	12.644 ± 1.145	36.296 ± 4.898	0.095
Ursodeoxycholic acid	56.366 ± 5.591	47.326 ± 5.895	61.695 ± 3.170	0.269
Glycine-conjugated				
Glycocholic acid	2.741 ± 0.534 ^a^	0.29 ± 0.0351 ^b^	0.393 ± 0.042 ^b^	<0.001
Glycochenodeoxycholic acid	2.217 ± 0.740 ^a^	0.1571 ± 0.0359 ^b^	0.190 ± 0.064 ^b^	<0.001
Glycodeoxycholic acid	4.089 ± 0.726 ^a^	1.882 ± 0.346 ^b^	2.109 ± 0.662 ^b^	0.019
Glycohyodeoxycholic acid	0.296 ± 0.034 ^b^	0.782 ± 0.140 ^a^	0.277 ± 0.060 ^b^	<0.001
Glycolithocholic acid	3.336 ± 0.776 ^b^	7.927 ± 1.55 ^a^	4.1414 ± 1.050 ^b^	<0.001
Taurine-conjugated				
Taurocholic acid	0.903 ± 0.010 ^a^	0.340 ± 0.0186 ^b^	0.435 ± 0.097 ^b^	<0.001
Taurochenodeoxycholic acid	0.276 ± 0.056 ^ab^	0.351 ± 0.055 ^a^	0.227 ± 0.010 ^ab^	0.107
Taurodeoxycholic acid	0.358 ± 0.017 ^ab^	0.412 ± 0.053 ^a^	0.287 ± 0.053 ^b^	<0.001
Taurohyodeoxycholic acid	0.254 ± 0.011 ^c^	1.244 ± 0.025 ^a^	1.211 ± 0.019 ^a^	<0.001
Taurolithocholic acid	0.129 ± 0.012 ^b^	0.320 ± 0.073 ^a^	0.124 ± 0.025 ^b^	<0.001

Values were expressed as mean ± SD (*n* = 8). ^a,b,c^ Means at the same row with different letters differed significantly at *p* < 0.05.

**Table 5:** Fecal short-chain fatty acid (SCFA) quantity and PH at week 6 in hamsters fed one of three diets: NCD, non-cholesterol diet; HCD, high-cholesterol diet; COS, HCD with addition of 5% chitosan-oligosaccharide (COS).

	NCD	HCD	COS	*p* Value
SCFAs (mg/day/hamster)				
Acetate	16.731 ±2.750 ^ab^	10.283 ± 1.776 ^c^	32.554 ± 7.933 ^a^	<0.001
Propionate	2.715 ± 0.271 ^ab^	1.215 ± 0.271 ^b^	2.179 ± 2.179 ^ab^	0.07
Butyrate	347.207 ± 20.788	327.517 ± 33.893	343.712 ± 23.534	0.785
Total SCFAs	366.654 ± 21.094	339.016 ± 34.119	378.446 ± 20.860	0.928
PH				
Fecal PH value	5.740 ± 0.176 ^c^	6.427 ± 0.056 ^a^	6.185 ± 0.190 ^b^	<0.001

Values were expressed as mean ± SD (*n* = 8). ^a,b,c^ Means at the same row with different letters differed significantly at *p* < 0.05.

## Data Availability

Not applicable.

## Supplementary Materials

The following supporting information can be downloaded at: https://www.mdpi.com/article/10.3390/nu15132923/s1, Figure S1: Rarefaction curves of Sobs index (A) and Shannon index (B) in hamsters fed one of the three diets: NCD, Non-cholesterol diet; HCD, high cholesterol diet; COS, HCD with addition of 5% chitosan-oligosaccharides (COS) (*n* = 6).
