# Supplementary material for: Marine Chitosan-Oligosaccharide Ameliorated Plasma Cholesterol in Hypercholesterolemic Hamsters by Modifying the Gut Microflora, Bile Acids, and Short-Chain Fatty Acids
Source: Nutrients. 2023 Jun 27;15(13):2923. doi: 10.3390/nu15132923 (PMC10346597; doi:10.3390/nu15132923)
Supplement: Supplementary file 1 [file nutrients-15-02923-s001.zip › nutrients-2416276-supplementary.pdf]

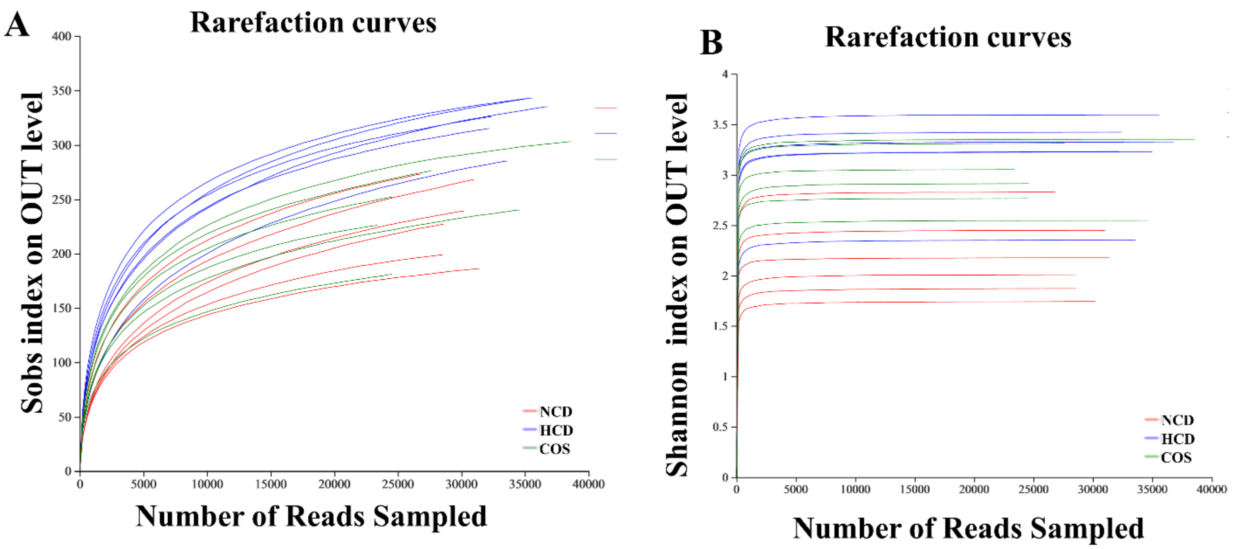

Supplementary Figure S1. Rarefaction curves of Sobs index (**A**) and Shannon index (**B**) in hamsters fed one of the three diets: NCD, Non-cholesterol diet; HCD, high cholesterol diet; COS, HCD with addition of 5% chitosan-oligosaccharides (COS). (*n* = 6).
